# Behavioral and Neural Correlates of Cognitive-Motor Interference during Multitasking in Young and Old Adults

**DOI:** 10.1155/2019/9478656

**Published:** 2019-07-01

**Authors:** Hannah Bohle, Jérôme Rimpel, Gesche Schauenburg, Arnd Gebel, Christine Stelzel, Stephan Heinzel, Michael Rapp, Urs Granacher

**Affiliations:** ^1^University of Potsdam, Research Focus Cognitive Sciences, Division of Social and Preventive Medicine, Am Neuen Palais 10, 14469 Potsdam, Germany; ^2^International Psychoanalytic University, Stromstraße 3b, 10555 Berlin, Germany; ^3^University of Potsdam, Research Focus Cognitive Sciences, Division of Training and Movement Science, Am Neuen Palais 10, 14469 Potsdam, Germany; ^4^Freie Universität Berlin, Clinical Psychology and Psychotherapy, Habelschwerdter Allee 45, 14195 Berlin, Germany

## Abstract

The concurrent performance of cognitive and postural tasks is particularly impaired in old adults and associated with an increased risk of falls. Biological aging of the cognitive and postural control system appears to be responsible for increased cognitive-motor interference effects. We examined neural and behavioral markers of motor-cognitive dual-task performance in young and old adults performing spatial one-back working memory single and dual tasks during semitandem stance. On the neural level, we used EEG to test for age-related modulations in the frequency domain related to cognitive-postural task load. Twenty-eight healthy young and 30 old adults participated in this study. The tasks included a postural single task, a cognitive-postural dual task, and a cognitive-postural triple task (cognitive dual-task with postural demands). Postural sway (i.e., total center of pressure displacements) was recorded in semistance position on an unstable surface that was placed on top of a force plate while performing cognitive tasks. Neural activation was recorded using a 64-channel mobile EEG system. EEG frequencies were attenuated by the baseline postural single-task condition and demarcated in nine Regions-of-Interest (ROIs), i.e., anterior, central, posterior, over the cortical midline, and both hemispheres. Our findings revealed impaired cognitive dual-task performance in old compared to young participants in the form of significantly lower cognitive performance in the triple-task condition. Furthermore, old adults compared with young adults showed significantly larger postural sway, especially in cognitive-postural task conditions. With respect to EEG frequencies, young compared to old participants showed significantly lower alpha-band activity in cognitive-cognitive-postural triple-task conditions compared with cognitive-postural dual tasks. In addition, with increasing task difficulty, we observed synchronized theta and delta frequencies, irrespective of age. Task-dependent alterations of the alpha frequency band were most pronounced over frontal and central ROIs, while alterations of the theta and delta frequency bands were found in frontal, central, and posterior ROIs. Theta and delta synchronization exhibited a decrease from anterior to posterior regions. For old adults, task difficulty was reflected by theta synchronization in the posterior ROI. For young adults, it was reflected by alpha desynchronization in bilateral anterior ROIs. In addition, we could not identify any effects of task difficulty and age on the beta frequency band. Our results shed light on age-related cognitive and postural declines and how they interact. Modulated alpha frequencies during high cognitive-postural task demands in young but not old adults might be reflective of a constrained neural adaptive potential in old adults. Future studies are needed to elucidate associations between the identified age-related performance decrements with task difficulty and changes in brain activity.

## 1. Introduction

In everyday life, the concurrent performance of postural and cognitive tasks is the norm rather than the exception. Waiting for the bus while talking to a friend represents an example for the simultaneous performance of a postural (i.e., standing) and cognitive (i.e., talking) task. Although postural control has traditionally been considered an automatic or reflex controlled task, recent research suggests that there are significant attentional demands. These demands vary depending on the postural task, the age of the individual, and their expertise in balance performance, resulting in different degrees of cognitive-motor interference [1]. Cognitive-postural multitasking is particularly affected in old adults and associated with an increased risk of falls [2]. Biological aging of both the cognitive and postural control systems appears to be responsible for higher cognitive-motor interference effects in old compared to young adults [3].

Postural control in situations with high compared to low postural demands (e.g., slips and trips during walking) afford higher order cognitive processing involving different brain structures (e.g., in the prefrontal cortex) [4–6]. While in young people, postural control is a seemingly effortless motor task, older adults' postural stability is challenged by biological aging of the sensory and neuromuscular systems as well as different brain areas (e.g., prefrontal cortex) [7–9]. Interference during the concurrent performance of postural and cognitive tasks in old age appears to result from the recruitment of overlapping cortical networks [10–13]. Furthermore, age-related decrements during the concurrent performance of cognitive and motor tasks are disproportionately greater than the additive age-related costs of performing the two tasks independently (dual-task costs) [14, 15]. In general, old adults show greater cognitive-motor interference than younger adults [16, 17]. However, with increasing postural demands, elderly seem to prioritize their attentional resources to control posture and to neglect cognitive performance [18, 19]. This “posture first” strategy is most likely chosen to lower the inherent risk of falling in potentially fall-threatening situations [16, 20].

Cognitive impairments in the form of reduced working memory capacity and associated neural changes have been reported for old compared with young adults [21–23]. As described within the framework of the “compensation-related utilization of neural circuits” hypothesis (CRUNCH; [24]), old adults seem to recruit neuronal resources at lower loads compared with young adults, leaving no adaptive reserve for higher loads.

In general, there is evidence that old compared to young adults show overall lower delta-, theta-, and alpha-band activities and higher beta-band activity [25–31].

Furthermore, it has been postulated that the delta response is related to signal detection and decision making, as the amplitude of the delta response is considerably increased in oddball experiments and in response to stimuli at the hearing threshold [32]. In the context of working memory studies, theta-band activity has been linked to working memory load and task difficulty [33–37]. In contrast, alpha-band activity was found to be suppressed by attentional processes or mental effort (e.g., [38–40]). Beta-band frequency is—amongst others—involved in movement control, and previously, it has been associated with inhibitory motor processes [31, 41–43].

To date, limited information is available on cognitive-motor interference in aging and underlying neural processes using electroencephalography (EEG). In a previous study, Beurskens et al. [44] examined the gait pattern and brain activity of young adults while concurrently performing a cognitive or motor interference task. Besides an impaired walking performance in dual- compared with single-task condition, the authors reported lower alpha-band activity at frontal and central electrodes while walking and concurrently performing a cognitive or motor interference task. In contrast, beta activity was increased during the secondary motor interference task at frontal sides, which was also linked to the additional recruitment of neural resources. In another study, Ozdemir et al. [45] compared cognitive and postural single and cognitive-postural dual-task performances in old and young adults at low and high task demands. Their participants performed a one-back (low cognitive task demand) or a two-back working memory task (high cognitive task demand) and stood on a fixed (low postural task demand) or a free-swinging platform (high postural task demand). Each task condition was either performed as a single task or in combination as a cognitive-postural dual task. According to Beurskens et al. [44], postural deficits were only found at high cognitive and high postural task demands. With regard to the underlying neural mechanisms, the authors reported a higher theta activity over frontal, central-frontal, and central areas with increased *cognitive* task difficulty as opposed to postural task difficulty. In addition, they found increased delta frequency synchronization over central-frontal, central, and central-parietal sites with increasing *postural* task demands. Furthermore, they found increased alpha frequency synchronization for increasing dual-tasking difficulty, i.e., for the one-back and two-back tasks on the sway platform. The increase of delta and alpha frequencies was more pronounced in young compared with old adults. The results indicate a general cognitive impairment in old compared to young adults. Furthermore, high postural task demands require more cognitive resources in old compared to young adults, resulting in additionally impaired cognitive performance. Nevertheless, the interpretation of the study is limited by a small sample size of 10 young and 9 old adults.

With reference to the relevant literature [44, 45], we aimed to expand previous findings using a larger sample size and a greater number of cortical Regions-of-Interest (ROIs) to examine behavioral and neural correlates of cognitive-motor interference during multitasking in young and old adults. In addition, we ran separate analyses for the midline electrodes Fz, Cz, and Pz to compare our results with those of Beurskens et al. ([44]). We expected that old adults are able to recruit additional neural resources to compensate for age-related working memory decline at low cognitive-postural task demands (CRUNCH; [24]). Thus, we hypothesized no differences in young compared to old adults with respect to cognitive performance at low cognitive-postural task difficulty. However, at high cognitive-postural task demands, we expected that neural compensatory mechanisms are limited in seniors resulting in both lower cognitive and postural performance in old compared to young adults [45]. Moreover, due to age-related changes in the sensorimotor and neuromuscular systems, we hypothesized that old compared to young adults show larger postural sway irrespective of the task [46]. With regard to the underlying neurophysiological effects, we hypothesized higher delta-, theta- and alpha-band activities as well as lower beta-band frequencies for young compared to old adults [25, 26, 29–31]. Furthermore, delta- and theta-band activities were expected to be higher and alpha-band activity to be lower with increased cognitive task difficulty in both age groups [27, 34, 36, 44, 45]. Due to an age-related cognitive decline, we assumed a synchronization in delta- and theta-band activities [45] and a desynchronization in alpha-band activity from low to high cognitive-postural task demands [44]. This effect was expected to be larger in young compared to old adults, as young adults appear to be able to recruit additional neural resources to maintain balance under high cognitive load [44]. Finally, we hypothesized lower beta-band power with increased cognitive-postural task demands in both age groups (e.g., [44]).

## 2. Materials and Methods

### 2.1. Participants

Overall, 31 healthy young (16 females) and 35 healthy old (17 females) adults were recruited to participate in this study. Three young and five old participants were excluded due to recording errors or EEG artifacts. Finally, 28 young (13 females; mean age 25.0, range 19-30 years, SD: 3.6) and 30 old (13 females, mean age 71.7, range 63–83 years, SD: 5.4) participants were enrolled in this study. None of the old adults showed signs of cognitive decrements or dementia, as assessed with the Mini Mental State Questionnaire [47]. The mean MMSE score amounted to 28.87 (SD: 1.14, minimum: 27) with the highest possible score being 30 and the cut-off for dementia usually being 23. Regarding medical treatment, 10 old participants (33%) declared that they were taking no drugs on a regular basis, 12 (40%) took medication for high blood pressure, 3 participants (10%) declared taking cholesterol-lowering drugs, and one participant was on mood-enhancing medication. In addition, 5 participants (12.8%) declared taking other nonpsychiatric drugs, such as Allopurinol against gout, eye drops, or nutritional supplements.

This experiment was part of a larger-scale study that involved functional magnetic resonance imaging and an intervention program for the old adults (data to be reported elsewhere). Young participants were recruited through a student mailing list at the University of Potsdam, Germany, whereas senior participants were primarily recruited through advertisements in regional newspapers in Potsdam and Berlin, Germany. All participants were healthy with no signs of neurological or psychiatric disorders. They did not suffer from hearing impairments, had normal or corrected-to-normal vision, and had experienced no falls during the past twelve months prior to the start of the study. This study was approved by the local ethics committee of the University of Potsdam and the study was conducted in accordance with the latest version of the Declaration of Helsinki. All participants were informed and provided their written consent prior to the start of the study. Reimbursement for both study groups was granted and comprised a minimum of 7.50 € per test hour. The cognitive performance data of 9 participants of the young group could not be considered in the analysis due to a technical failure during the recording of the vocal responses. These participants were not considered in the analysis of the working memory task.

### 2.2. Design and Procedure

**Figure 1:**
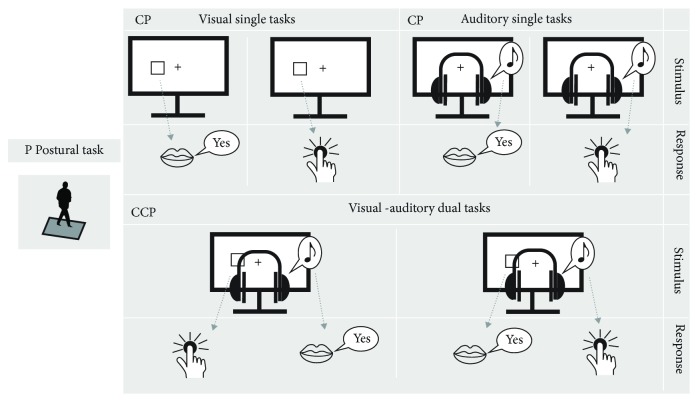
Illustration of the task design: all tasks were performed on a force plate in semitandem stance. The experiments consisted of three different task types: (1) during the “Postural Task” (P), subjects fixated on a visual dynamic stimulus; (2) during the “Cognitive-Postural Task” (CP), participants performed a visual or auditory one-back task; (3) during the “Cognitive-Cognitive-Postural Task” (CCP), subjects performed an auditory and a visual one-back task in conjunction.

**Figure 2:**
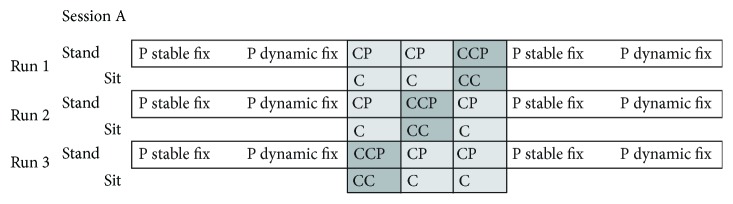
The figure presents one session, including three runs. Each run consists of a standing condition and a sitting condition. In the standing condition, participants were either exposed to a stable or a dynamic fixation condition. The stable fixation condition and the sitting conditions were not analyzed in the present study. The standing condition consisted of seven task blocks and the sitting condition of three task blocks. After completing one run with visual-manual and auditory-vocal response mappings, another run with visual-vocal and auditory-manual response mappings was conducted. The order of runs was pseudorandomized across participants. P: postural task; C: cognitive task; CP: cognitive-postural task; CCP: cognitive-cognitive postural task.

All tests took place at the biomechanics laboratory (University of Potsdam, Germany) on two separate test days. The time between the two test days ranged from one to four weeks. On the *first test day*, general hearing ability, visual ability, and general cognitive functioning were assessed. In addition, a battery of more specific neuropsychological and motor tests (e.g., Digit Span, Trail Making A and B, Stopping Task, and Timed Up and Go Test) was conducted. At the end of this test session, participants were familiarized with the experimental task that was scheduled for the second test day. On the *second test day*, leg dominance was examined using Coren's lateral preference inventory [48]. Subsequently, participants were prepared for EEG recordings. For the postural task, participants stood on a balance pad to measure Center of Pressure (CoP) displacements. The balance pad was placed on top of a force plate to increase task difficulty through an unstable surface. Participants performed three different task types (see Sections 2.3.1, 2.3.2, and 2.3.3 for more detail). In the “Postural Single Task” (P), participants had to fixate on a dynamic visual stimulus. During the “Cognitive Task” (C), participants performed the spatial one-back task without additional postural demands. The results of C were not further analyzed. In the “Cognitive-Postural Dual Task” (CP), participants performed a spatial one-back task with either auditory or visual stimuli combined with either manual or vocal responses in addition to the postural task. In the “Cognitive-Cognitive-Postural Triple Task” (CCP), participants conducted a visual-auditory cognitive dual task, which combined the visual and auditory spatial one-back task and the postural task (see Figures 1 and 2). Total CoP displacements and EEG recordings were synchronized during the task. We chose a within-subject block design which comprised two sessions. Within each session, the mapping between stimulus modalities and response modalities was kept constant. Session order was counterbalanced between participants. Data were obtained in a sitting and in a standing condition. Due to our specific research question, we focused data analysis on cognitive-motor interference conditions. Data during sitting was not further analyzed here. Each session comprised three runs, where each run consisted of three counterbalanced task conditions (CCP; visual CP; and auditory CP) (see Figure 2). The postural task (P) was presented at the beginning and the end of each run. The order of conditions within the run was kept the same across participants. Before the start of each of the experimental sessions, participants conducted one practice block for each of the CP tasks and two practice blocks for the CCP task. Participants were encouraged to rest between task blocks, especially when performing in semitandem stance position to provide sufficient relaxation and recovery for their legs between the task blocks and to avoid cramps due to unfamiliar physical work load. The two experimental sessions were separated by a break of several minutes to allow recovery until participants felt ready to perform the second part. Each session lasted approximately 30-40 min.

### 2.3. Experimental Paradigm

We used presentation software (https://www.neurobs.com/) to present visual and auditory task stimuli and to record manual and vocal responses. Using a self-developed MATLAB tool, vocal data were analyzed offline to identify correct and false responses and RTs. Successful validation of the custom-made tool [49] was obtained by manual coding of vocal responses (Cohens Kappa = .94, *p* < .001). Performance in cognitive tasks was calculated as *p*(hit) − *p*(false alarm). This approach was chosen to consider the number of correct responses as well as the number of incorrect responses, i.e., when no response was needed. Dual-task costs of cognitive performance were calculated using the following formula: ([CCP − CP]/CP∗100). According to Doumas et al. [20], dual-task costs express the effects of the additional costs imposed in individual-task performance in a dual-task setting.

A priori power analyses with G∗Power (Version 3.1.9.4, University of Kiel, Germany) [50] using two groups and a repeated measures analysis of variance (rmANOVA) design with 2 measurements and within-between interactions yielded a total sample size of *N* = 68 (effect size *f* = 0.4, *α* = 0.05), with an actual power of 0.90 (critical *F* value = 3.99). Effect size was estimated using previously published work on the effects of different unstable surfaces and bases of support on cortical activity (i.e., spectral power of theta frequency band) in young adults [51]

#### 2.3.1. Postural Single Task

Participants were instructed to stand with the arms hanging loose to the sides of the body in semitandem stance on a balance pad with the dominant leg placed posterior to the nondominant leg. Moreover, participants had to keep their head straight and their gaze fixed to a stable or a dynamic visual stimulus. In the dynamic condition, a fixation cross and an ampersand symbol (font size: 54) were alternatively presented in the middle of the screen. The presentation times were matched to the working memory task (500 ms ampersand, 1500 ms fixation cross). Here, we only report data of the dynamic stimulus condition, as pilot data revealed higher CoP displacements in the dynamic condition and thus indicated larger task difficulty. Data during sitting were not further analyzed here, also because we had not included a postural single-task condition (fixation) for a direct comparison with the standing condition.

#### 2.3.2. Cognitive-Postural Dual Task (CP)

Input stimuli were visual or auditory with either manual or vocal response output requirements (see Figure 1). Visual input stimuli were squares which were displayed in one of six possible positions (left or right side; bottom, middle, and up) presented in the center of the monitor screen (white squares on black screen; presentation times: 500 ms). Auditory input stimuli comprised three different tones (200, 450, and 900 Hz), which were presented via headphones either to the left or to the right ear. Participants were instructed to respond as fast and correct as possible when a target stimulus appeared. A target stimulus was defined as a stimulus which was identical to the previously presented stimulus, i.e., when the same stimulus appeared in the same position. Participants had to respond to visual and auditory stimuli with either a vocal response, i.e., by saying “yes” in German, or with a manual response, i.e., a button press on a device which was held in the right hand. One single-task block lasted 33 s and included 16 stimuli with five target stimuli. In the *CP* task condition, participants had to process either the visual or the auditory task.

#### 2.3.3. Cognitive-Cognitive-Postural Triple Task (CCP)

In the CCP task condition, participants were asked to process auditory and visual one-back tasks (CP) simultaneously. One CCP block lasted 33 s and included 16 stimuli in each modality with five target stimuli.

### 2.4. Measurement of Postural Control

Postural sway (i.e., total CoP displacements) was assessed using a one-dimensional force plate (Leonardo 105 Mechanograph®; Novotec Medical GmbH, Pforzheim, Germany) at a sampling rate of 800 Hz. A balance pad (Airex®) was placed on the plate. During testing, participants had to stand as still as possible for 33 s in semitandem stance. For this purpose, the dominant foot was placed behind the nondominant foot. Total CoP displacements (mm) were computed using CoP displacements in mediolateral and anterior-posterior directions. A test duration of 33 s was chosen to comply with the cognitive task requirements and to achieve acceptable reliability of postural stability measurements (LeClair and Riach, 1996). Test blocks were excluded from further data analyses if participants lost their balance.

### 2.5. EEG Data Acquisition

**Figure 3:**
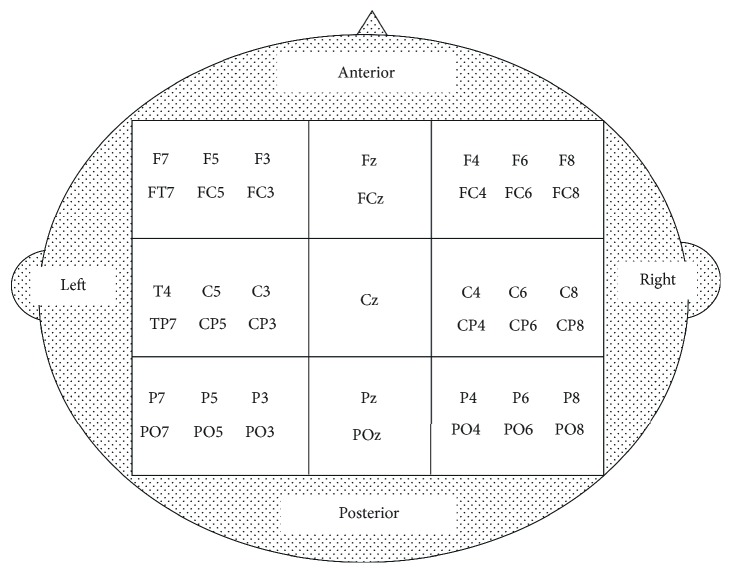
Approximate placement for the electrodes included in the Region-of-Interest (ROI) analysis for rmANOVAs according to [53]. The rectangles indicate the levels used to demarcate the nine ROIs. Unlike [53], the CPz electrode was used as a reference electrode in our analysis and therefore not included in the central midline ROI.

We used a 64-channel mobile EEG system (Advanced Neuro Technology (ANT), Enschede, Netherlands). One electrode was placed under the right eye to measure horizontal eye movements. The cap was positioned on the participants' scalp according to the International 10-20 standard system. Channels were referenced to the CPz electrode and resistance was kept below 5 k*Ω* for all electrodes. During EEG testing, we controlled for movement artifacts by asking our participants to avoid bodily movements (e.g., jaw clenching and eye, head, and arm movements). The EEG signal was recorded at a sampling rate of 1024 Hz using eego™ software (Advanced Neuro Technology, Enschede, Netherlands). For later offline analysis, BrainVision Analyzer (Version 2.1., Brain Products GmbH, Munich, Germany) was used with a 0.5–45 Hz bandpass filter (time constant: 0.33 s, slope: 48 dB/octave) and an ocular correction filter as provided by BrainVision Analyzer (Brain Products GmbH, Gilching, Germany). High-frequency bands such as the gamma frequency band were omitted with the filter due to its known confoundation with muscle activity (see [52]). Artifacts were semiautomatically rejected (gradient: <35 *μ*V; amplitude range: -100 to 100 *μ*V). Subsequently, EEG data was segmented into 1 s epochs, analyzed using spectral analysis (FFT), and averaged across 33 s. Average voltage activity was exported for delta- (0.5–<4 Hz), theta- (4–7.5 Hz), alpha- (8–12 Hz), and beta-band (13–30 Hz) frequencies. EEG frequencies delta, theta, alpha, and beta were examined with ROI analyses. In accordance with Dube et al. [53], we demarcated nine ROIs (anterior midline (Fz, FCz), central midline (Cz), posterior midline (Pz, POz), anterior left (F7, F5, F3, FT7, FC5, and FC3), central left (C3, C5, T7, CP3, CP5, and TP7), posterior left (P7, P5, P3, PO7, PO5, and PO3), anterior right (F4, F6, F8, FC4, FC6, and FT8), central right (C4, C6, T8, CP4, CP6, and TP8), and posterior right (P8, P4, P6, PO4, PO6, and PO8)) (Figure 3). Unlike Dube et al. [53], CPz was used as a reference electrode in our analysis, and therefore, this electrode was not included in the central midline ROI. In accordance with Beurskens et al. [44], all additional midline analyses included Fz, Cz, and Pz.

### 2.6. Statistical Analyses

#### 2.6.1. Cognitive Performance

*Cognitive performance* was assessed as mean *p*(hit) − *p*(false alarm) per condition. Repeated measures analysis of variances (ANOVA) were applied including the within-subject factor “*task*” (CP, CCP) and the between-subject factor “*group*” (young, old).

#### 2.6.2. CoP

*CoP* was calculated by means of an ANOVA including the within-subject factor “*task*” (P, CP, and CCP) and the between-subject factor “*group*” (young, old).

#### 2.6.3. EEG Data and ROI Analyses

EEG data were analyzed using separate 9 × 2 × 2 rmANOVAs with the within-subject factors ROI (9 ROIs) and task (dual-task CP relative to fixation/triple-task CCP relative to fixation) as well as the between-subject factor age group (young/old). In the ROI analyses, the posture condition (P) was used as a baseline condition. The frequency bands of the dual-task (CP) and the triple-task (CCP) conditions were attenuated with the frequency bands of the posture (P) condition, using the following formulae: CP_relative_to_fix = ([CP − P/P]∗100) and CCP_relative_to_fix = ([CCP − P]/P)∗100. Significant main effects for ROI and ROI × task, ROI × age, and ROI × task × age interactions were further analyzed in terms of laterality and anterior-posterior effects. For this, we ran a separate analysis for the within-subject factors anterior − central − posterior (3) × laterality (2) × task (2) and the between-subject factor age group (2). That way, we were able to trace back ROI effects to potential laterality effects and/or effects along the anterior-posterior axis. If these effects were not significant, more subtle ROI differences must be assumed which should be tested with spatially higher resolved methods. Two participants had to be excluded from this ROI analysis due to incomplete data sets for the included electrodes. As the three midline ROIs were analyzed separately in accordance with Beurskens et al. (see the following discussion), these were excluded from these post hoc analyses. The results of this analysis are presented in Table 1 for all four frequency bands as well as all the main effects and interactions, if they were included in the analysis.

Third, for the analysis focusing on the *midline* (see [44]), we calculated 3 × 3 × 2 ANOVAs for each frequency band including the factors “*task*” (P, CP, and CCP), “*electrode*” (Fz, Cz, and Pz), and the between-subject factor “*age group*” (young, old).

For post hoc comparisons, ANOVAs and *t*-tests were computed. Effect sizes (Cohen's *d* or partial *η*^2^) were reported for all analyses. If sphericity was violated, the Greenhouse-Geisser correction was applied. The significance level was set at *α* = 5%. All statistical analyses were processed using IBM SPSS Statistics, Version 25.0.

## 3. Results

### 3.1. Cognitive Performance

**Figure 4:**
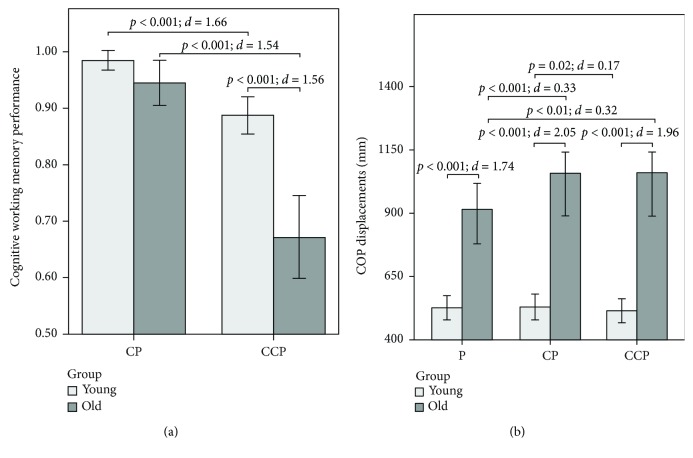
(a) Cognitive working memory performance *p*(hit) − *p*(false alarm) for old (*n* = 30) and young (*n* = 27) participants. Whiskers represent a standard deviation. P: postural task; CP: cognitive-postural task; CCP: cognitive-cognitive-postural task. (b) CoP displacements (mm) for old (*n* = 30) and young (*n* = 27) participants. Whiskers represent a standard deviation. P: postural task; CP: cognitive-postural task; CCP: cognitive-cognitive-postural task.

Cognitive performance data are illustrated in Figure 4(a). Irrespective of the age group, performance was better in the CP compared to the CCP condition (*F*(1, 47) = 83.35, *p* < .001, *d* = 2.67). Young compared to old adults were significantly better in their cognitive performance (*F*(1, 47) = 14.56, *p* < .001, *d* = 1.12). A significant task × group interaction (*F*(1, 47) = 18.82, *p* < .001, *d* = 1.28) indicated better performance in young compared to old adults in the CCP (*t*(39.11) = 5.30, *p* < .001, *d* = 1.56) but not in the CP condition (*t*(38.71) = 1.80, *p* = .08, *d* = 0.53).

### 3.2. Postural Performance

CoP data are illustrated in Figure 4(b). Old compared to young adults showed larger total CoP displacements in all task conditions (*F*(1, 55) = 52.63, *p* < .001, *d* = 1.96). Moreover, a significant task × group interaction (*F*(1.43, 78.61) = 10.81, *p* < .001, *d* = .87) indicated an increase in total CoP displacements in old adults from P to CP (*t*(29) = 5.33, *p* < .001, *d* = 0.33) and from P to CCP (*t*(29) = 3.42, *p* < .01, *d* = 0.32) but not from CP to CCP (*t*(29) = .08, *p* = .94, *d* < 0.01). In contrast, in young adults, we observed a decrease in total CoP displacements from CP to CCP (*t*(26) = 2.53, *p* = .02, *d* = 0.17). There were no significant changes from P to CP or from P to CPP (all *t*'s <1.32, *p*'s >.15, and *d*'s <0.10).

### 3.3. EEG Frequency Bands

To address the age variability within the old adults, we divided the old age group with a median split (median: 72 years) into a “younger old age subgroup” (63-71 years, *N* = 14) and an “older old age subgroup” (72-83 years, *N* = 15) and compared the spectral power of the delta, theta, alpha, and beta frequency bands between the subgroups. However, we did *not* find a significant difference within the old age group regarding the subgroups neither for the delta frequency band (*F*(1, 28) = 2.80, *p* = .106) nor for the theta frequency band (*F*(1, 28) = 3.08, *p* = .090), the alpha frequency band (*F*(1, 28) = .579, *p* = .453), or the beta frequency band (*F*(1, 28) = .709, *p* = .407).

#### 3.3.1. Delta Band

**Figure 5:**
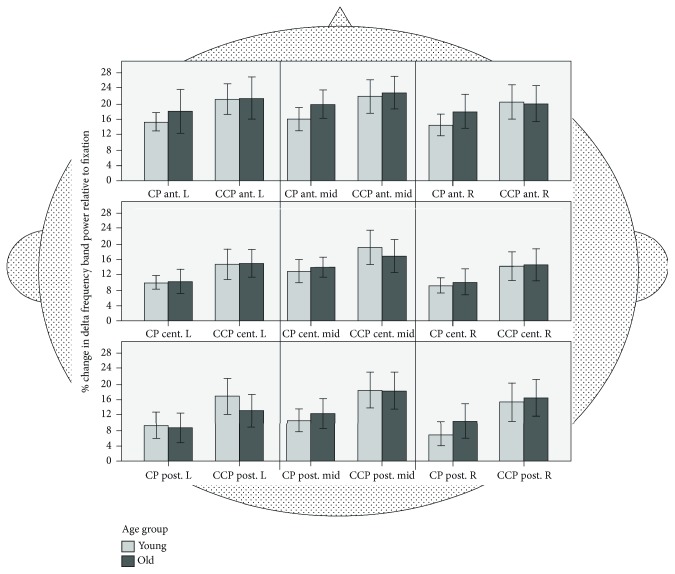
Mean delta-band frequency activity for young (*n* = 28) and old (*n* = 30) adults across nine ROIs: anterior, central, posterior, midline, left, and right. Frequency bands were calculated relative to the fixation condition (P). CP: cognitive-postural task; CCP: cognitive-cognitive-postural task.

*(1) Delta ROI Analyses*. Delta activity differed between the nine ROIs (*F*(8, 432) = 16.19, *p* < .001, *η*^2^_*p*_ = .231) and for task conditions (*F*(1, 54) = 51.27, *p* < .001, *ŋ*^2^_*p*_ = .487). Higher delta frequencies were found for the triple-task CCP compared to CP (see Figure 5). Age group did not significantly influence the delta frequency band (*F*(1, 54) = .121, *p* = .729, *ŋ*^2^_*p*_ = .002).

A significant ROI × task interaction was also found for delta (*F*(8, 432) = 3.84, *p* < .001, *ŋ*^2^_*p*_ = .066). To further specify the general ROI effect as well as the ROI × task interaction pattern, we ran an additional ANOVA with factors anterior − central − posterior (3) × laterality (2) × task (2). For this analysis, the three midline ROIs were excluded. This analysis (see Table 1) revealed general topographical differences in the delta band in the anterior-posterior axis with greater delta activity in anterior ROIs compared to central (*t*(55) = 7.61, *p* < .001) and posterior (*t*(55) = 5.41, *p* < .001) ROIs. In addition, differences between CCP and CP across age groups were larger in posterior compared to anterior (*t*(55) = 3.04, *p* = .004) and central (*t*(55) = 2.58, *p* = .013) ROIs. These differences were triggered mostly by effects in the right hemisphere, as indicated by the significant task × laterality × ACP interaction (see Table 1).

*(2) Delta Midline Analysis*. Also in the midline electrodes, young compared to old participants showed generally higher delta-band activities (*F*(1, 54) = 5.03, *p* < .05, *ŋ*^2^_*p*_ = .085). A main effect of task (*F*(2, 108) = 157.13, *p* < .001, *ŋ*^2^_*p*_ = .744) indicated a higher theta-band activity with increased task difficulty, irrespective of age. A main effect of electrode (*F*(2, 108) = 512.6, *p* < .001, *ŋ*^2^_*p*_ = .905) indicated differences between them, with a decrease from anterior to posterior (Fz: mean (*M*) = 8.21, standard error (SE) = .026; Cz: *M* = 4.21, SE = .018; Pz: *M* = 3.96, SE = .015). The delta frequency band for the electrodes differed between the age groups (electrode × group: (*F*(2, 108) = 12.69, *p* < .001, *ŋ*^2^_*p*_ = .190). A one-way ANOVA for electrode (3) by group (2) revealed that the group difference was only present in the Fz electrode (*F*(1, 54) = 10.61, *p* < .05, *ŋ*^2^_*p*_ = .016) but not in Cz (*F*(1, 54) = 2.07, *p* = .156, *ŋ*^2^_*p*_ = .016) or Pz (*F*(1, 54) = .867, *p* = .356, *ŋ*^2^_*p*_ = .037) for all tasks. Higher values were found in the young group. Thus, midline analyses for delta-band activity revealed a general age-related increase in activity in young participants at the midanterior electrode during task performance.

#### 3.3.2. Theta Band

**Figure 6:**
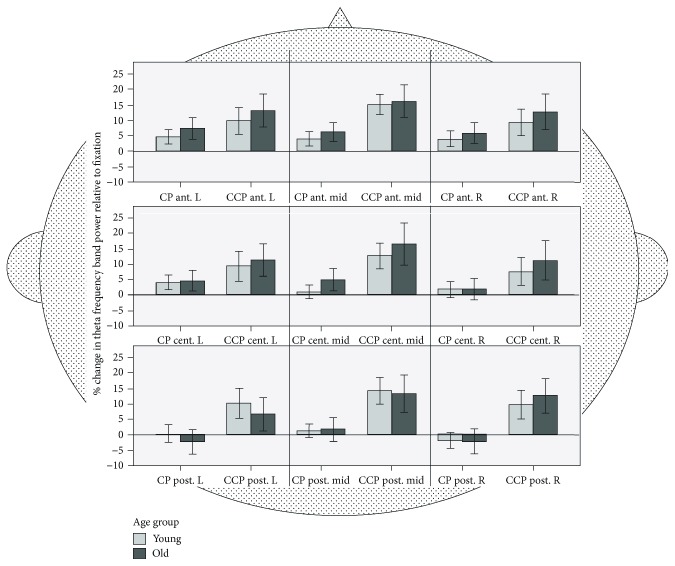
Mean theta-band frequency activity of the young (*n* = 28) and old (*n* = 30) groups for nine ROIs: anterior, central, posterior, midline, left, and right. Frequency bands are calculated relative to fixation (P). CP: cognitive-postural task; CCP: cognitive-cognitive-postural task.

*(1) Theta ROI- Analyses*. Theta activity differed for the nine ROIs (*F*(8, 432) = 12.34, *p* < .001, *ŋ*^2^_*p*_ = .186). Theta-band frequency differed between tasks (*F*(1, 54) = 79.06, *p* < .001, *ŋ*^2^_*p*_ = .59), with a higher activity for CCP compared to CP (see Figure 6). There was no significant main effect of age group, showing that there was no general difference between young and old adults in the theta band (*F*(1, 54) = .302, *p* = .302, *ŋ*^2^_*p*_ = .006). However, significant ROI × group interactions (*F*(8, 432) = 2.74, *p* = .006, *ŋ*^2^_*p*_ = .048), ROI × task interactions (*F*(8, 432) = 20.9, *p* < .001, *ŋ*^2^_*p*_ = .279), and ROI × task × group interactions were found (*F*(8, 432) = 2.14, *p* < .001, *ŋ*^2^_*p*_ = .038), indicating an age-related modulation of theta activity. This effect is modulated by task and brain region.

To elucidate the topography of ROI effects, we set up a follow-up rmANOVA with within-subject factors anterior − central − posterior (3) × laterality (2) × task (2) and the between-subject factor age group (2). This analysis (see Table 1) revealed general topographical differences in the theta band in the anterior-posterior axis with greater theta activity in anterior ROIs compared to posterior (*t*(55) = 2.58, *p* = .012) and central compared to posterior (*t*(55) = 4.38, *p* < .001) ROIs.

In addition, differences between CCP and CP across age groups were generally more pronounced in the right hemisphere (laterality × task interaction) and in posterior ROIs compared to anterior (*t*(55) = 6.04, *p* < .001) and central (*t*(55) = 3.33, *p* < .001) ROIs. Importantly, these effects were partly age dependent—CCP-CP differences between hemispheres were only present in old (*t*(28)=4.72, *p* < .001) but not in young adults, and additionally mostly pronounced in posterior ROIs (*t*(54) = 4.05, *p* < .001) as indicated by the age group × laterality × ACP interaction.

*(2) Theta Midline Analysis*. Young compared to old participants showed generally higher theta-band activity in the midline electrodes (*F*(1, 56) = 11.29, *p* < .01, *d* = .91). A main effect of task (*F*(1.43, 80.29) = 63.79, *p* < .001, *d* = 2.12) indicated a higher theta-band activity with increased task difficulty (P < CP (*t*(57) = 6.37, *p* < .001, *d* = .18); CP < CCP (*t*(57) = 8.01, *p* < .001, *d* = .26)), irrespective of age.

#### 3.3.3. Alpha Band

**Figure 7:**
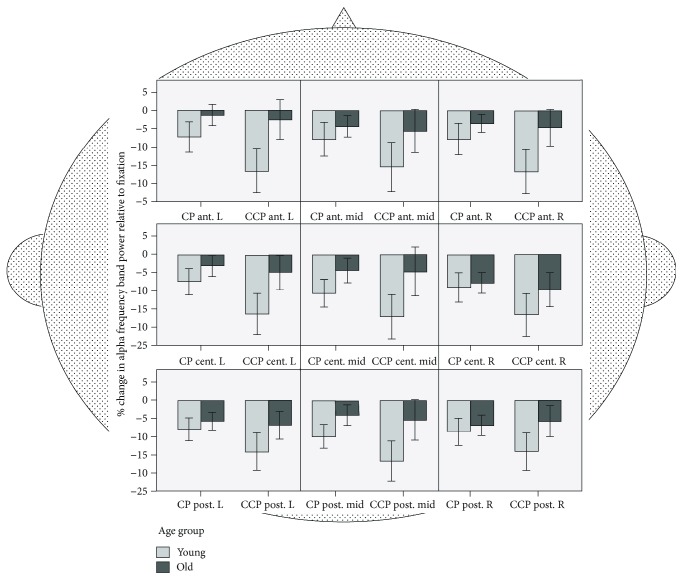
Mean alpha-band frequency activity for the young (*n* = 28) and old (*n* = 30) groups for nine ROIs: anterior, central, posterior, midline, left, and right. Frequency bands are calculated relative to fixation (P). CP: cognitive-postural task; CCP: cognitive-cognitive-postural task.

*(1) Alpha ROI Analyses*. Alpha-band activity differed between the nine ROIs (*F*(8, 432) = 3.2, *p* = .002, *ŋ*^2^_*p*_ = .056). Alpha-band frequency differed between tasks (*F*(1, 54) = 14.53, *p* < .001, *ŋ*^2^_*p*_ = .212), with lower activity for CCP compared to CP (see Figure 7). Alpha frequency band generally differed between the two age groups (*F*(1, 54) = 6.69, *p* = .012, *ŋ*^2^_*p*_ = .110), with lower alpha-band activity in young participants.

Significant ROI × group interactions (*F*(8, 432) = 3.39, *p* = .001, *ŋ*^2^_*p*_ = .059), task × group interaction*s* (*F*(1, 54) = 8.53, *p* = .005, *ŋ*^2^_*p*_ = .136), and ROI × task interactions (*F*(8, 432) = 3.67, *p* < .001, *ŋ*^2^_*p*_ = .64) were found. The follow-up ANOVA included the within-subject factors anterior − central − posterior (3) × laterality (2) × task (2) and the between-subject factor age group (2). This analysis revealed generally lower alpha activity across age groups in the right compared to the left hemisphere and in central compared to anterior ROIs (*t*(55) = 3.28, *p* = .002). Task-independent hemispheric differences between age groups were present in central ROIs only (*t*(55) = 2.60, *p* = .012). CP-CCP differences were generally greater in young compared to old participants (*t*(54) = 3.21, *p* = .002) and greater in anterior ROIs compared to posterior ROIs (*t*(55) = 3.33, *p* = .002) as well as in central compared to posterior ROIs (*t*(55) = 4.10, *p* < .001). The post hoc tests of the four-way interaction indicated that greater CP-CCP differences for young compared to old participants were present in all ROIs except in the left posterior ROI.

*(2) Alpha Midline Analysis*. Young compared to old participants showed higher alpha-band activities in the midline electrodes Fz, Cz, and Pz (*F*(1, 56) = 10.26, *p* < .01, *d* = 0.87). This effect was most pronounced for the Fz electrode as indicated by a significant electrode × group interaction (*F*(1.19, 66.53) = 11.18, *p* < .001, *d* = 0.91). A significant main effect of task (*F*(1.23, 68.86) = 35.30, *p* < .001, *d* = 1.60) reflects a decrease in alpha-band activity with increased task difficulty (P > CP (*t*(57) = 5.54, *p* < .001, *d* = 0.15); CP > CCP (*t*(57) = 4.77, *p* < .001, *d* = 0.13)). A significant main effect of electrode (*F*(1.19, 66.53) = 213.65, *p* < .001, *d* = 3.88) indicates highest alpha-band activities at frontal sides and decreased activities at central and posterior electrodes (Fz > Cz (*t*(57) = 14.52, *p* < .001, *d* = .68); Cz = Pz (*t*(57) = .127, *p* = .90, *d* < 0.01)). A significant task × group interaction (*F*(1.23, 68.86) = 6.68, *p* < .01, *d* = 0.70) indicates greater decreases in alpha-band activity with increased task difficulty in young (*t*(27) = 4.96, *p* < .001, *d* = 0.38) compared to old adults (*t*(29) = 3.32, *p* < .01, *d* = 0.21).

Of note, in the midline analysis, the frequency band was *not* calculated as deviations from the fixation-baseline condition P. Instead, the postural-task P, the cognitive-postural dual-task CP, and the triple-task CCP were considered separately, i.e., in absolute values. Therefore, young compared to old adults showed higher frequency band values. On the other hand, when frequency bands are represented as deviations from postural-task P, as presented above, alpha frequencies are lower for the young group compared to the old group.

#### 3.3.4. Beta Band

*(1) Beta ROI Analysis*. Beta activity differed for the nine ROIs (*F*(8, 432) = 3.421, *p* < .001, *ŋ*^2^_*p*_ = .06) and for ROI × task (*F*(8, 432) = 5.738, *p* < .001, *ŋ*^2^_*p*_ = .096), but not for task (*F*(1, 54) = 2.20, *p* = .144, *ŋ*^2^_*p*_ = .039) and not between age groups (*F*(1, 1) = 1.36, *p* = .249, *ŋ*^2^_*p*_ = .25).

**Figure 8:**
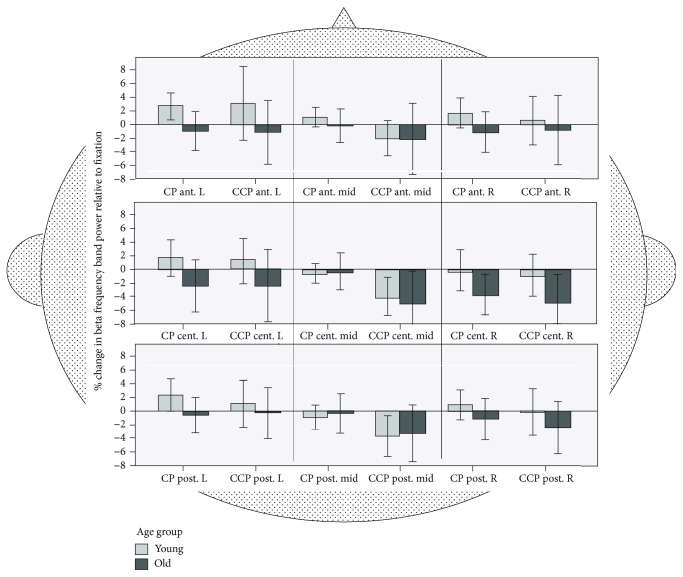
Mean beta-band frequency activity for young (*n* = 28) and old (*n* = 30) adults for nine ROIs: anterior, central, posterior, midline, left, and right. Frequency bands are calculated relative to fixation (P). CP: cognitive-postural task; CCP: cognitive-cognitive-postural task.

Given that we identified the effects of ROIs and ROI × task but not for age, we ran a follow-up analysis only for the within-subject factors anterior − central − posterior (3) × laterality (2) × task (2). This analysis (see Figure 8) indicated generally lower beta-band activity for the right compared to the left hemisphere but no task-specific differences.

*(2) Beta- Midline Analysis*. The analysis of the midline gave a significant main effect of task (*F*(1.35, 75.49) = 7.16, *ε* = .67, *p* < .01, *d* = 0.70), which is indicative of a decrease in beta-band activity with increased task difficulty (P > CP (*t*(57) = 16.88, *p* < .001, *d* = .71); CP > CCP (*t*(29) = 3.12, *p* < .01, *d* = .11)). A significant electrode × group interaction (*F*(1.31, 73.33) = 5.541, *ε* = .66, *p* = .01, *d* = 0.63) shows age-related differences related to electrode localization. For further analysis, a three factor ANOVA analysis was computed with the within-subject factor “task” and “electrode” (Fz, Cz, and Pz) and the between-subject factor “*age group*.” Results revealed greater beta-band activity in old compared to young participants at Fz (*F*(1, 56) = 5.16, *p* = .03, *d* = 0.59) but not at Cz or Pz (all *F*'s <2.89, all *p*'s >.09, all *d*'s <0.46).

## 4. Discussion

This study aimed at identifying performance and neural correlates in cognitive-motor interference tasks in young and old adults. Therefore, young and old individuals were asked to perform a semitandem stance on a balance pad while concurrently processing visual and/or auditory working memory tasks. In line with the CRUNCH model, we expected that old adults recruit additional neuronal resources at low but not high task demands to compensate for age-related cognitive decline. However, at high task demands, old adults lack an adaptive reserve which may result in performance decline.

In general, old compared to young participants showed larger postural sway. In particular, old adults showed higher CoP displacements for the cognitive-postural dual-task (CP) and triple-task (CCP) conditions compared to the postural single-task condition (P). The finding that there was no difference in total CoP displacements under CP and CCP conditions in old adults seems congruent with the idea of dynamic adaptations depending on the task load. Note that in our previous study, relative costs in postural sway (not absolute displacement) of old adults was enhanced in triple- (CCP) compared to dual-task (CP) conditions, but only under certain additional task requirements such as the type of input-output modality mappings, which were not part of the present study [17]. This suggests, that various factors (e.g., age and task type) might affect behavioral adaptations to task requirements in cognitive-postural multitasking.

We further showed an age-related decline in cognitive performance at high cognitive-postural task demands (CCP). In accordance with our hypothesis, the identified ceiling effects in both age groups indicate good cognitive performance during the realization of the cognitive-postural task condition (CP). However, marked performance declines were noted in both groups during the triple-task condition (CCP). This drop in performance was greater in old compared to young adults, as indicated by larger effect sizes. Our findings show that the applied task manipulation successfully discriminated between easy and challenging cognitive tasks and that it is reflective of a high strain, particularly in old participants.

Regarding age-related changes in neural activation, we performed a whole brain ROI analysis and analyzed EEG frequencies relative to the postural condition (P). This approach takes age-related differences in baseline activity as well as topographical differences into account. Accordingly, delta, theta, alpha, and beta frequencies were not presented in absolute values, but as deviations relative to the fixation or baseline condition P (see Figures 5–8). These analyses revealed a circumscribed pattern of results, with age-related differences in the correlates of cognitive-postural multitasking being most pronounced in the theta and alpha frequency bands.

In terms of topography, delta, theta, and alpha frequency bands were generally more synchronized in anterior brain regions compared to posterior regions. With increasing task demands, delta as well as theta frequency bands were higher (CCP > CP) and alpha bands lower (CP > CCP) for both age groups. Interestingly, for the alpha frequency band, the young group exhibited a greater decrease for CCP compared to CP. The task-dependent alpha band decrease for the young group was more pronounced in anterior and central regions.

In accordance with our hypothesis regarding the midline electrodes (see [44]), we found higher delta and theta frequency bands, as well as lower alpha and beta frequency bands for young compared to old participants [25, 26, 30, 31]. In this study, the postural single task (P), the cognitive-postural dual task (CP), and the cognitive-cognitive-postural triple task (CCP) were analyzed separately, which is why data were not adjusted for potential baseline between group differences.

### 4.1. Delta Frequency Effects

In line with our hypothesis, irrespective of age, delta-band frequency was higher in the triple (CCP) compared with the dual-task condition (CP). This indicates delta-band synchronization with increasing cognitive task demands (P < CP < CCP). These findings were consistent for the ROI analyses as well as the analyses of the midline electrodes.

Our results are in accordance with findings from Ozdemir et al. [45]. These authors reported increased delta activity in both age groups during the most challenging postural task condition, especially over the frontal, central-frontal, and central regions. The authors concluded that delta activity seems to be most sensitive to postural challenges, as opposed to working memory loads. More specifically, delta frequency seems to increase only when dual tasking includes a challenging postural condition on a sway platform but not on a fixed (stable) platform. Of note, delta frequencies were not affected by cognitive load.

According to Babiloni et al. [54], neural synchronization of delta frequencies can be interpreted as a general neurophysiological mechanism that sets in as task demands increase. Consequently, information processing within distributed functional neural networks have to be enhanced. These authors suggested that low frequencies may govern a long-range coordination of distant brain regions, for instance, a multioscillatory functional network between high-order frontal motor areas [54].

Our subsequent analyses revealed higher delta-band synchronization in frontal regions compared to central or posterior regions. In addition, differences between the cognitive-postural dual task (CP) and the cognitive-cognitive-postural triple task (CCP) were more pronounced in posterior compared to anterior and central regions. However, differences were only present in the right hemisphere, with higher delta-band frequency power. We found a stronger synchronization for the more demanding triple-task condition (CCP) compared to the dual-task (CP) condition, irrespective of age.

Concerning laterality effects, Handel et al. [55] reported increased delta amplitudes primarily in parietal regions with increasing motion coherence in a magnetoencephalography study. These changes in delta amplitudes were only observed in hemispheres contralateral to the location of the visual stimulus presentation. Notably, the visual stimulus was always visible in both visual hemifields. Therefore, the authors suggested that selective attention increased with motion coherence. This again resulted in higher delta amplitudes in contralateral parietal areas to the location of stimuli in which the respective visual information is processed. Furthermore, there is evidence from a PET study that tonal stimuli are processed in the right hemisphere if an additional task (e.g., an oddball task) is performed simultaneously. No lateralization was observed if the tonal stimuli was presented alone [56]. Moreover, Sininger and Bhatara [57] reported prioritized activation of the right hemisphere, when tonal stimuli were presented in three different frequencies to healthy young adults. As we used visual stimuli as well as auditory stimuli to evoke motor and vocal responses, effects for laterality and task condition might be attributed to attentional processes related to preferred hemispheric processing. Taken together and in view of the literature, our findings of increased delta frequency band power in the right hemisphere between posterior and anterior as well as central ROIs were dependent on task complexity. The findings might be explained by increased selective attention on visual and auditory perception.

### 4.2. Theta Frequency Effects

Theta-band frequency differed according to task conditions. Higher activities were found in CCP compared with CP (see Figure 6). In addition, we observed topographical differences in theta-band activity in the anterior-posterior axis with the highest theta activity in anterior ROIs, compared with central and posterior ROIs.

The increase in the theta band for CCP compared to CP is in line with our hypothesis, as it reflects a higher task difficulty in working memory manipulations ([33–37], Scharinger, Soutschek, Schubert and Gerjets, 2017). Apparently, the relevance of frontal theta power in memory tasks has previously been shown to be reliable Assenza et al. [58]. Also, according to Ozdemir et al. [45], theta-band EEG activity seems to be more responsive to working memory performance during dual-task conditions with challenging two-back cognitive working memory tasks, as opposed to one-back working memory tasks. Increased theta-band activity was found in the young and the old age groups over frontal, central-frontal, and central cortices.

Interestingly, our subsequent analyses (see Table 1) revealed age- and task-related differences in theta oscillations. The observed differences between CCP and CP in young and old adults were more pronounced in posterior ROIs compared with anterior and central ROIs. Of note, CCP-CP differences in posterior ROIs were found in old adults only. According to Kardos et al. [30], frontal-midline theta oscillations observed during task execution seem to reflect general sustained attention, whereas the frontal-midline theta power modulation with varying memory task demands might reflect the effect of an active maintenance process. Furthermore, Kardos et al. [30] suggest that frontal functions may implement top-down processes that can modify the dynamical activity in other brain regions. This might be illustrated in the form of coordinating a distributed information processing functional network in parietal brain regions [30]. Following this argument, presumably top-down mechanisms in seniors activated additional neuronal resources in posterior regions to successfully process the most difficult postural-postural-cognitive triple task. This could explain, why we observed higher theta-band synchronization for the triple-task (CCP) condition in the posterior right ROI for old but not young adults.

Also for the midline electrodes, we expected a synchronization in the theta-band activity [45] from low to high cognitive-postural task demands due to age-related cognitive decline. In line with our hypothesis, the theta-band frequencies were increased in both age groups compared to the fixation condition. Theta-band activity increased with increasing task difficulty compared to fixation (P), i.e., for the dual-task (CP) and triple-task (P < CP < CCP) conditions. For the midline, young adults showed higher theta-band frequencies than seniors. According to Assenza et al. [58], the enhanced presence of the slow theta oscillation may suggest a network disassembly. In addition, it could also be interpreted as a sign of plasticity in the context of neural network reorganization. This process could be enhanced in young adults which is illustrated in higher theta-band frequency.

### 4.3. Alpha Frequency Effects

Alpha-band activity is primarily observed over visual and attentional network areas. Alpha-band activity is negatively related to visual perception, suggesting that the inhibition of external visual input is helping performance of internal memory tasks. This observation has led to the hypothesis that alpha-band activity may shape functional network architecture through inhibiting task-irrelevant areas [58].

Accordingly, in our study, task-specific as well as age-related differences were most consistent in the alpha band. Young adults showed a pronounced effect for task, i.e., the alpha band decreased for the triple-task CCP compared to CP which is indicative of a decrease with cognitive load (CP > CCP). Interestingly, the task effect is mainly due to the effects of the young adults, as seniors did not show differences in the alpha band with increasing cognitive load (CP = CCP). The ROI analysis confirms the task- and age-specific effects for nearly the whole brain. Alpha suppression was observed during the performance of the demanding triple-task (CCP) condition compared with the dual-task condition. This was found in all ROIs, except for the left posterior ROI.

These findings are in accordance with results from Beurskens et al. [44] who reported a reduced alpha-band frequency during dual-task compared with single-task walking in young participants. These results indicate a selective mobilization of additional effort in the demanding triple task in young participants.

The desynchronization of the alpha-band frequency with increasing levels of cognitive load in young adults is a common finding ([44, 58]; Scharinger et al. 2017; [59, 60]). For instance, Zhavoronkova et al. [60] examined young healthy participants, standing on a stabilography platform. Common center of pressure (CCP) movements in the lateral plane were used as a simple motor task and CCP movements in different directions served as a more complex motor task, while simple or complex calculation tasks served as cognitive tasks. In their study, both components of the cognitive-postural dual tasks were performed worse as compared to the single tasks. In the cognitive-motor dual tasks, EEG alpha desynchronization was more pronounced than during the performance of separate tasks. The authors state that a decrease in the coherence of the *α*1 band in the frontal areas appears as a “zone of interference.” According to McEvoy et al. [38], only young adults seem to show a decrease in alpha power with increased task difficulty. The authors compared age-related working memory differences in the EEG frequency band of young, middle-aged, and old adults.

Taken together, alterations in the alpha band might represent the additional recruitment of neural resources in young compared to old adults in order to cope with higher task demands. Specifically, differential alpha-band frequencies in young adults might reflect an adaptation to cognitively more demanding settings. In order to compensate the age-related cognitive performance decline, old compared to young adults recruit additional neural resources at low task demands. However, in old age, adaptive neural reserves can be recruited at higher task demands which only leads to an age-related decline in cognitive performance (CRUNCH; [24]). In particular, it might reflect the ability of the young adults to suppress distracting or irrelevant external information for the purpose of successfully focusing on the memory task [58]. In other words, in old participants, neural adaptive processes might be diminished which is reflected in higher performance decrements together with a lack of modulating activities in relevant frequencies.

### 4.4. Beta Frequency Effects

In contrast to our expectations, on the whole brain level, the beta-band frequency was neither modulated by age nor by task differences. However, our findings are in line with Ozdemir et al. [45] who could not find a modulation of the beta band either for both single- and dual-task balance conditions. In these experiments, participants were asked to stand as still as possible during upright erect stance on a fixed platform surface or a sway platform surface.

To be able to compare the results of our study with findings from Beurskens et al. [44], we ran a separate analysis on the midline electrodes. The analysis of the midline indeed showed a task effect, with desynchronizing or lower beta frequencies with higher working memory load (P > CP > CCP). Interestingly, Beurskens et al. [44] found a beta-band *synchronization* over the midline electrodes for their motor interference task compared to dual-task walking and for dual-task walking compared to single-task walking. For the motor interference task, participants had to walk while balancing a stick with interlocked rings at the end. In the dual-task situation, individuals had to walk and simultaneously subtract numbers. Therefore, the motor interference tasks as well as dual-task walking were rather challenging compared with our study in which participants were standing still on a force plate.

In summary, according to Assenza et al. [58], the beta band seems to be a prominent rhythm of the corticospinal system, which is assumed to track the efficient flow of motor information between the cortex and the periphery [58]. In particular, our findings suggest that beta-band oscillations seem to *synchronize* for rather challenging motor interference tasks and cognitive-motor tasks which involve walking at increasing physical demands. However, for easier cognitive-motor tasks involving postural control they seem to *desynchronize* over midline cortical areas with increasing working memory load. Finally, on a whole brain level, beta-band frequencies appear not to be modulated by postural control or cognitive-postural dual tasking.

### 4.5. Conclusions and Limitations

In general, the present study revealed greater cognitive-postural interference in old compared to young adults, which was reflected in age-specific neural modulations. Old adults showed greater postural instability and impaired cognitive performance compared with young adults, particularly at high task loads. Overall, the neural activation patterns reflect these higher task demands in the cognitive-postural dual- and triple-task conditions, either by an increase in delta-, beta-, and theta-band or a decrease in alpha-band frequency.

The modulation of the alpha, beta, and theta frequency bands seem to reflect an increasing working memory load. For instance, Scharinger et al. (2017) reported concurrent decreases in alpha and beta frequency band power at parietal electrodes (i.e., the so-called event-related desynchronization (ERD)) during the performance of *n*-back working memory tasks. This was accompanied by increased theta power at frontal electrodes (i.e., the so-called event-related synchronization (ERS)). Delta frequency modulations can be explained by high cognitive-postural task demands [45].

Even though the present study revealed novel findings with respect to the neural correlates of cognitive-motor interference, our results should be considered within the scope of some limitations. First, the main limitation of the present study is the lack of an increasing postural task difficulty during our experimental setup. More specifically, participants were only assessed during upright erect standing in semitandem stance but not during bipedal or single-leg stance or even during sitting. Hence, due to a missing baseline control condition with low postural (i.e., sitting) and no cognitive demands, our conclusions are limited with regard to the underlying neural processes of postural control. In addition, due to our specific experimental setup, findings apply for semistance but not for walking conditions. Second, due to a recording error of the vocal response during the working memory task, we had to exclude nine participants from our analysis of the working memory task. Third, the present study focused on the comparison of old versus young adults. It would be interesting to apply the same paradigm with additional samples, such as middle-aged individuals or patient groups. Due to these limitations, results from this study should be interpreted with caution.

Overall and in line with the previous literature, we found evidence for behavioral and neural impairments in cognitive-motor multitasking. Particularly, old compared with young participants showed a pronounced decline in cognitive performance at high task demands (CCP). Furthermore, the old group showed higher postural sway during cognitive-postural (CP, CCP) compared with single-mode postural tasks (P). This indicates that old adults follow a posture-first strategy at the expense of cognitive performance. On the contrary, the young group showed a decrease at high (CCP) compared to low task demands (P, CP). In terms of neural correlates, we found higher theta- and alpha-band activities in young compared with old adults. In addition, delta-, theta-, and alpha-band activities varied as a function of the cognitive-postural task demand. Young compared with old adults showed higher adaptive potential in the alpha band to respond to increasing difficulty levels. This indicates an age-related decline in neural adaptive processes if task difficulty changes and affords flexible modulation of neural processes.

Taken together, findings from this study showed an involvement of attentional processes during postural control in young versus old adults. We were also able to show age-, ROI-, and frequency-specific adaptations in brain activity with increasing task difficulty.

## Figures and Tables

**Table 1:** Statistical results of follow-up ROI analysis.

	Delta	Theta	Alpha	Beta
Main effects				
Task (i) CP vs. CCP	*F*(1, 55) = 50.43, *p* < .001, *ŋ*^2^_*p*_ = .478 CCP > CP	*F*(1, 54) = 60.23, *p* < .001, *ŋ*^2^_*p*_ = .527 CCP > CP	*F*(1, 54) = 17.52, *p* < .001, *ŋ*^2^_*p*_ = .245 CP > CCP	*F*(1, 55) = .272, *p* < .60, *ŋ*^2^_*p*_ = .005
Age group (i) Young (Y) vs. old (O)	No age-related effect	*F*(1, 54) = .211, *p* = .648, *ŋ*^2^_*p*_ = .004	*F*(1, 54) = 6.23, *p* = .016, *ŋ*^2^_*p*_ = .103 O > Y	No age-related effect
Laterality (i) Left (L) vs. right (R)	*F*(1, 55) = .196, *p* = .66, *ŋ*^2^_*p*_ = .004	*F*(1, 54) = 1.26, *p* = .27, *ŋ*^2^_*p*_ = .023	*F*(1, 54) = 9.71, *p* = .003, *ŋ*^2^_*p*_ = .152 L > R	*F*(1, 55) = 7.58, *p* = .008, *ŋ*^2^_*p*_ = .121 L > R
ACP—anterior (A) vs. central (C) vs. posterior (P)	*F*(2, 110) = 27.35, *p* < .001, *ŋ*^2^_*p*_ = .332 A > C, A > P, C = P	*F*(2, 108) = 14.4, *p* < .001, *ŋ*^2^_*p*_ = .211 A = C, A > P, C > P	*F*(2, 108) = 3.40, *p* = .037, *ŋ*^2^_*p*_ = .059 A > C, A = P, C = P	*F*(2, 110) = 2.48, *p* < .09, *ŋ*^2^_*p*_ = .043
2-Way interactions				
Task × age	No age-related effect	*F*(1, 54) = .586, *p* = .45, *ŋ*^2^_*p*_ = .011	*F*(1, 54) = 10.3, *p* = .002, *ŋ*^2^_*p*_ = .160 CP-CCP differences: Y < O	No age-related effect
Laterality × age	No age-related effect	*F*(1, 54) = 2.5, *p* = .112, *ŋ*^2^_*p*_ = .045	*F*(1, 54) = 3.87, *p* = .054, *ŋ*^2^_*p*_ = .670	No age-related effect
ACP × age	No age-related effect	*F*(2,108) = .586, *p* = .45, *ŋ*^2^_*p*_ = .011	*F*(2, 108) = .6.00, *p* = .007, *ŋ*^2^_*p*_ = .100 A: O > Y; C: O > Y; P: O = Y	No age-related effect
Laterality × task	*F*(1, 55) = .309, *p* = .58, *ŋ*^2^_*p*_ = .006	*F*(1, 54) = 23.13, *p* < .001, *ŋ*^2^_*p*_ = .300 CP-CCP differences: R > L	*F*(1, 54) = 2.19, *p* < .145, *ŋ*^2^_*p*_ = .038	*F*(1, 55) = 1.90, *p* = .173, *ŋ*^2^_*p*_ = .034
ACP × task	*F*(2, 110) = 6.40, *p* = .013, *ŋ*^2^_*p*_ = .104 CCP-CP differences: A = C, P > A, P > C,	*F*(2, 108) = 32.74, *p* < .001, *ŋ*^2^_*p*_ = .377 CCP-CP differences: A = C, A > P, C > P	*F*(2, 108) = 10.98, *p* < .001, *ŋ*^2^_*p*_ = .169 CCP-CP differences: A = C, A > P, C > P	*F*(2, 110) = .392, *p* = .676, *ŋ*^2^_*p*_ = .007
Laterality × ACP	*F*(2, 110) = .729, *p* = .49, *ŋ*^2^_*p*_ = .130	*F*(2, 108) = 23.13, *p* < .001, *ŋ*^2^_*p*_ = .300 L: A > C, A > P, C > P R: A > C, A > P, C = P	*F*(2, 108) = 6.47, *p* = .002, *ŋ*^2^_*p*_ = .107 L: A = C, A = P, C = P R: A > C, A = P, P > C	*F*(2, 110) = .535, *p* = .59, *ŋ*^2^_*p*_ = .010
3-Way interactions				
Task × age × laterality	No age-related effect	*F*(1, 54) = 8.11, *p* = .006, *ŋ*^2^_*p*_ = .131 CCP-CP differences: Young: L = R Old: R > L	*F*(1, 54) = .11, *p* = .92, *ŋ*^2^_*p*_ < .001	No age-related effect
Task × age × ACP	No age-related effect	*F*(2,108) = .656, *p* = .52, *ŋ*^2^_*p*_ = .012	*F*(2,110) = 1.99, *p* = .142, *ŋ*^2^_*p*_ = .035	No age-related effect
Age × laterality × ACP	No age-related effect	*F*(2, 108) = 9.77, *p* < .001, *ŋ*^2^_*p*_ = .153 L-R differences: A: Y = O; C: Y = O; P: O > Y	*F*(2, 108) = 3.69, *p* = .028, *ŋ*^2^_*p*_ = .064 L-R differences: A: O = Y; C: O > Y; P: O = Y	No age-related effect
Task × laterality × ACP	*F*(2, 110) = 4.24, *p* = .044, *ŋ*^2^_*p*_ = .072 CCP-CP differences: L: A = C, A = P, C = P R: A = C, P > A, P > C	*F*(2, 108) = 9.36, *p* < .001, *ŋ*^2^_*p*_ = .148 CCP-CP differences: L: A = C, P > A, P > C R: C > A, P > A, P > C	*F*(2,108) = 2.16, *p* < .120, *ŋ*^2^_*p*_ = .038	*F*(2,110) = .14, *p* = .99, *ŋ*^2^_*p*_ < .001
4-Way interactions				
Task × age × laterality × ACP	No age-related effect	*F*(2,108) = 2.85, *p* = .062, *ŋ*^2^_*p*_ = .05	*F*(2,108) = 3.24, *p* = .043, *ŋ*^2^_*p*_ = .057 CCP-CP differences: L_A: Y < O L_C: Y < O L_P: Y = O R_A: Y < O R_C: Y < O R_P: Y < O	No age-related effect

## Data Availability

The data used to support the findings of this study are available from the corresponding author upon request.
